# Teacher Self-Efficacy and Well-Being: The Mediating Role of Satisfaction with Students, Colleagues, and Parents

**DOI:** 10.3390/ijerph23020150

**Published:** 2026-01-25

**Authors:** Federica Marcedula, Giacomo Angelini, Caterina Fiorilli

**Affiliations:** Department of Human Science, LUMSA University, 00193 Rome, Italy; marcedulaf@gmail.com (F.M.); fiorilli@lumsa.it (C.F.)

**Keywords:** teacher well-being, teacher self-efficacy, job satisfaction, school relationships, mental health

## Abstract

**Highlights:**

**Public health relevance—how does this work relate to a public health issue?**

- Teacher stress and burnout represent growing public health concerns with significant implications for mental health.
- Identifying protective factors can help sustain a healthy and effective educational workforce.

**Public health significance—why is this work of significance to public health?**

- The study shows that self-efficacy and relational satisfaction are key contributors to teachers’ psychological well-being.
- Enhancing these resources may reduce stress-related outcomes and improve school functioning.

**Public health implications—what are the key implications or messages for practitioners, policy makers and/or researchers in public health?**

- Programs strengthening self-efficacy and school relationships can promote teacher well-being and resilience.
- Policymakers and school leaders should prioritize interventions that support positive relational climates in schools.

**Abstract:**

The recent literature has increasingly drawn attention to the role of teachers’ personal and relational resources in managing stress and sustaining their well-being. In this study, we examined how self-efficacy and satisfaction in key school relationships contribute to teachers’ psychological health. A sample of 339 Italian teachers (*M_age_* = 49.7, *SD* = 9.26; 85.5% female) completed measures assessing their self-efficacy, satisfaction in relationships with students, colleagues, and parents, and their overall well-being. We tested a parallel mediation model to explore whether these three forms of relational satisfaction helped explain the link between self-efficacy and well-being. The analyses indicated that higher self-efficacy was associated with greater satisfaction across all relational domains, as well as with better well-being. Moreover, satisfaction with students, colleagues, and parents each emerged as a significant mediator, while the direct effect of self-efficacy remained significant, suggesting a pattern of partial mediation. Taken together, these findings underscore how both individual competencies and everyday relational experiences contribute to teachers’ well-being, pointing to the value of interventions that strengthen self-efficacy and enhance the quality of relationships within the school context.

## 1. Introduction

### 1.1. Well-Being

Recent years have seen a significant increase in research on teacher well-being [1], highlighting that teachers are particularly susceptible to work-related stress, psychological challenges, and depression [2,3,4]. Indeed, teaching is a stressful, intensive, and complex profession because it involves numerous stressors that increase the risk of burnout [5].

Among the many stressors, some are particularly relevant, including workplace changes such as larger and more diverse classes, updated school curricula, and the introduction of new teaching and learning technologies, low salaries and increased workload due to staff shortages [6]. Also, the change in society, and the economy and politics of today are contributing to this. According to the International Labor Organization, teachers can get sick, and have breathing problems, muscle pain and voice problems because they work in noisy and unhealthy environments [7]. Moreover, teachers are tasked with fostering students’ personal growth and supporting their academic performance [8]. In recent years, this responsibility has become increasingly demanding, not only due to growing pressures from administrators, students, and parents, which further intensifies their workload [9]. This work is considered a “helping profession”, and this makes it difficult to find a balance between one’s own resources and the work demands [6,10].

The psychological well-being of teachers is important to investigate because it is a profession at risk of burnout and work-related stress and a stressed teacher might have negative effects on the health and performance of students [11]. In fact, emotionally and socially competent teachers can promote positive relationships among students and with students, contributing to the growth of a sense of autonomy and competence in class, stimulating ‘love’ towards study, and reducing the risk of developing behavioral and emotional difficulties in their students [12]. For these reasons, much of the psychological–educational research is aimed at identifying the dynamics and factors that can influence the well-being of teachers in school. Several studies have indicated that the well-being of workers, including teachers, can be prevented and protected by the role of psychosocial/personal and organizational factors [13].

### 1.2. Self-Efficacy

Among the personal variables, self-efficacy has proved to be a protective factor for teachers. Self-efficacy refers to individuals’ beliefs about their capacity to organize and carry out the actions needed to handle specific situations [14]. In the educational context, teacher self-efficacy encompasses not only confidence in delivering instructional content, but also the perceived ability to manage classroom dynamics, foster student motivation, and collaborate effectively with families throughout the learning process [15,16]. The study by Zee and Koomen [17] on teacher self-efficacy and its influence on well-being analyzed how self-efficacy has demonstrated positive correlations with factors that influence teachers’ psychological well-being, including personal success, job satisfaction, and commitment. Teachers with a high level of self-efficacy tended to be more engaged in their work, were more likely to employ advanced teaching methods, and showed greater willingness to work with students as well as to build positive relationships with principals and colleagues, and they tended to report lower levels of burnout, stress, and anxiety compared to teachers with low self-efficacy [18].

### 1.3. Satisfaction

Teachers are not isolated in their roles; they are integral members of a professional community that includes colleagues and the principal, as well as students and families. Consequently, to work effectively and achieve a sense of personal satisfaction, educators must collaborate with their peers in managing the workload and personal/organizational resources [19]. According to the theoretical framework put forth by [20], the job satisfaction of teachers is significantly influenced by the quality of the relational environment they work in. The authors highlight how teachers perceive support, trust, and cooperation within their school community plays a crucial role in their overall satisfaction with their professional lives. Within this framework, three key relational areas stand out as particularly important. First, satisfaction with colleagues refers to how much teachers feel they can collaborate, encourage one another, and share responsibilities with their peers. Next, satisfaction with students relates to how teachers view their students’ behavior, engagement, and responsiveness in the classroom, factors that greatly affect their daily emotional experiences. Lastly, satisfaction with parents reflects how teachers perceive their interactions and communication with families, including the level of involvement and support they receive in the educational process. Together, these aspects illustrate the complex nature of relational satisfaction in schools and emphasize how various interpersonal relationships can either support or undermine teachers’ overall well-being. Numerous studies have shown that job satisfaction is positively linked to emotional, psychological, and social well-being. For instance, job satisfaction reduces the chance of changing jobs and fosters positive emotions towards teachers and students [21]. As outlined in the research conducted by Skaalvik and Skaalvik [22], the satisfaction of secondary school teachers is generally determined by three key factors: work organization (including working conditions and organizational autonomy), some cognitive aspects (such as self-efficacy) and various affective dimensions (emotions and stress). Therefore, work satisfaction was also studied as an important mediator between working conditions/personality and welfare outcomes [23].

Many recent studies have shown that relationships play a key role in teachers’ work and that the satisfaction from positive relationships with coworkers, parents and students can mitigate some of the negative effects of teaching [24]. In addition to these studies, further evidence supports this idea.

There is broad agreement among researchers about the connections that teachers have with their students (e.g., [25]), identifying these interactions as the primary source of work-related stress for educators. Challenges in classroom management often stem from negative relationships, which can contribute significantly to stress and burnout as teachers progress through their careers [26]. Teachers who are highly motivated and satisfied with their profession, according to a new study by Day and Gu [27], are also rewarded by the students’ good performance and their good relationships.

Social relations with one’s colleagues within the workplace are another dimension of how they affect employee job satisfaction. Similarly, research by Ghenghesh [28] has shown that the nature of teachers’ relationships with their colleagues plays a crucial role in determining their job satisfaction.

Finally, reflecting contemporary perspectives on the social elements of teacher roles, the model also incorporated parental satisfaction as a third dimension. Extensive research [29] have investigated the significance of parental involvement in relation to children’s academic success, indicating that families should be fully integrated into school activities. A strong correlation was identified between the nature of parental involvement and school performance, especially in terms of promotion and maintenance of communication about school events, homework and encouragement of reading practices.

### 1.4. Rationale of the Study

The reviewed literature has shown that higher levels of self-efficacy are associated with greater motivation, lower emotional exhaustion, and more positive indicators of psychological well-being [17,30,31]. At the same time, the quality of school relationships represents a key factor for teachers’ emotional and professional functioning, influencing job satisfaction, classroom management, and perceptions of organizational support [32]. Despite this solid evidence, most studies have examined these dimensions independently. Research on self-efficacy has predominantly focused on individual motivational processes, whereas studies on school relationships have typically analyzed single actors (e.g., students or colleagues) without considering the combined influence of the main relational domains that characterize teachers’ daily work. In the Italian context, several studies have shown that teacher well-being is shaped by both personal factors and relational or organizational characteristics, including collaboration among colleagues, relationships with families, and the management of student behavior [33]. However, no study to date has simultaneously investigated satisfaction with students, colleagues, and parents as parallel mediators of the relationship between self-efficacy and well-being. The present study positions itself within this research gap by proposing an integrated model that examines three potentially crucial relational domains in parallel. In doing so, it aims to clarify how personal and social resources combine to sustain the psychological well-being of Italian teachers, providing an original contribution both theoretically and practically.

### 1.5. Aims and Hypotheses

Based on the theoretical framework described, this study aims to analyze how and to what extent relationship satisfaction (with colleagues, parents and students) mediates the relationship between self-efficacy and well-being. Considering the three sub-scales of satisfaction, we implemented a parallel mediation model to analyze the relationships between self-efficacy and teachers’ well-being with the three sub-scales of satisfaction as mediators.

However, due to the absence of previous studies specifically focused on the three dimensions of satisfaction, we formulated the following research question:

In our study, we want to identify how the dimensions of satisfaction (with students, colleagues and parents) influence the relationship between self-efficacy and well-being in samples of teachers, and, for this, we have established the following hypotheses:

**Hypothesis** **1:** *Teachers’ satisfaction, respectively, with students, parents and colleagues, is positively related to both self-efficacy and well-being (H1).*

**Hypothesis** **2:** *Teachers’ satisfaction, respectively, with students, parents and colleagues, positively mediates the relationship between self-efficacy and well-being (H2).*

## 2. Method

### 2.1. Participants

The study involved 339 Italian teachers (age range = 20–67; *Mage* = 49.7, *SD* = 9.26), with 290 of them being women (85.5%). Participants worked in either primary school (grades 1–5; ages 6–11; 51.6%) or upper secondary school (grades 9–13; ages 14–19; 48.4%). Eligibility criteria were being an Italian teacher and providing voluntary informed consent to participate. The inclusion criteria for the study required that participants be Italian teachers who voluntarily agreed to take part.

### 2.2. Procedure

The method of conducting this study in Italy involved using a cross-sectional descriptive design and convenience sampling. Data were collected through an online survey run on Google Forms. Participants were recruited during training project meetings held in schools. Before completing the survey, participants were informed of the research objectives and informed consent for data collection and processing procedures. Data were collected anonymously, participants joined voluntarily, freely and without any compensation. No data were excluded, and no missing values were detected. This study was conducted in accordance with the privacy and informed consent requirements of the current Italian legislation (DL-196/2003). The research project was accepted by the LUMSA University’s Scientific Research Ethics Committee (CERS), and the study was conducted within the framework of the Helsinki Declaration.

### 2.3. Instrumentation

#### 2.3.1. Teacher Self-Efficacy Scale (SAED-SF)

The Teacher Self-Efficacy Scale-Short Form (SAED-SF [15]; Italian version [34]) includes 12 items measuring self-efficacy. All items were scored on a nine-point Likert scale, ranging from *‘nothing’* (1) to *‘a great deal’* (9). In this study, SAED-SF has demonstrated good psychometric properties with Cronbach’s α (α = 0.815) and McDonald’s ω (ω = 0.819).

#### 2.3.2. Teacher Job Satisfaction Scale (TJSS-9)

The Teacher Job Satisfaction Scale (TJSS-9 [20]) consists of 3 sub-scales measuring satisfaction with co-workers (3 items; e.g., *‘The extent to which your co-workers encourage you and support you in your work’*), satisfaction with parents (3 items; *‘Your overall level of satisfaction with parents where you work’*) and satisfaction with students’ behaviors (3 items; e.g., *‘Your satisfaction with the behavior of students in your school’*). The TJSS-9 includes 9 items divided into three for each sub-scale. All items were scored on a five-point Likert scale, ranging from ‘I am highly dissatisfied with this aspect of the school’ (1) to *‘I am highly satisfied with this aspect of the school*’ (5). Responses were summed and averaged for each sub-scale; scoring ranged between one and five. The total TJSS-9 score is used to assess job satisfaction. The independent scores on the three sub-scales provide more information about teacher satisfaction as a complex and diverse phenomenon depending on the personal connection that is established. TJSS has demonstrated satisfactory psychometric properties with adequate internal consistency and good Cronbach’s α and McDonald’s ω for all three sub-scales (α coworkers = 0.879, ω coworkers = 0.880; α students = 0.885, ω students = 0.888; α parents = 0.759, ω parents = 0.769).

#### 2.3.3. Mental Health Continuum-Short Form (MHC-SF)

The Mental Health Continuum-Short Form (MHC-SF [35]; Italian version [36]) includes 14 items measuring well-being. All items were scored on a six-point Likert scale, ranging from ‘none of the time’ (0) to ‘all of the time’ (5). The total MHC-SF scores are in the range of 0 to 70, with higher scores indicating better mental health. In this study MHC-SF has demonstrated good psychometric properties with Cronbach’s α (α = 0.904) and McDonald’s ω (ω = 0.908).

#### 2.3.4. Socio-Demographic Information

A series of questions were also administered that investigated the socio-demographic field, i.e., gender, age, and education level.

## 3. Analysis

**Figure 1:**
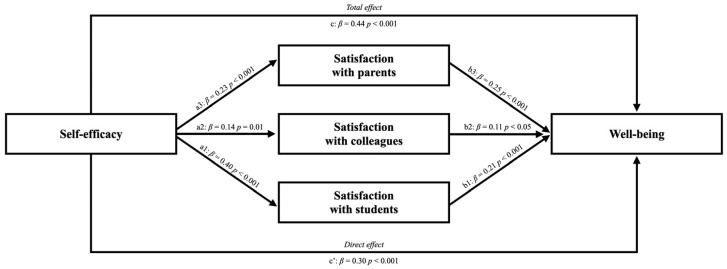
The relationship between self-efficacy and well-being, with different sub-scales of satisfaction as parallel mediators: a model of parallel mediation.

The SPSS statistical software (v.27.0; IBM Corp., Armonk, NY, USA) was used to analyze the collected data. The means and standard deviations for all scales were calculated. The *p*-value < 0.05 was considered a significant deviation from the normality of the distribution. Pearson’s correlation analysis evaluated the associations between the variables under study (efficacy, satisfaction and well-being). To evaluate the effect of efficacy on well-being and explore the role of different sub-scales of satisfaction, a parallel mediation model using the macro-program PROCESS 4.0 (New York, NY, USA) [37] was tested. Concerning the model specification, self-efficacy was the predictor, well-being was the outcome, and the three sub-scales of satisfaction were the mediator variables. The hypothesized model is shown in Figure 1. Specifically, Figure 1 shows the effect of self-efficacy on satisfaction with students (a1), satisfaction with coworkers (a2), satisfaction with parents (a3). Furthermore, Figure 1 shows the effect of satisfaction with students (b1), satisfaction with coworkers (b2), satisfaction with parents (b3) on well-being. Finally, Figure 1 also shows the total effect of self-efficacy on well-being (c) and the direct effect of self-efficacy on well-being (c′). The 95% confidence interval (CI) was calculated for each regression coefficient included in the models. Finally, the statistical relevance of the indirect effects was verified by performing the bootstrap technique for each of the 5000 bootstrapped samples within 95% of the confidence interval.

## 4. Results

Table 1 shows the measures’ means (M), standard deviations (SD), and Pearson’s correlation matrix.

The results highlighted that well-being and efficacy were significantly and positively correlated with each other (r = 0.439, *p* < 0.001). Furthermore, all associations were significant and positive of self-efficacy with satisfaction with students (r = 0.402, *p* < 0.001), satisfaction with coworkers (r = 0.140, *p* < 0.01), satisfaction with parents (r = 0.225, *p* < 0.001). Similarly, there was a significant and positive correlation between well-being and all sub-scales of satisfaction, i.e., with students (r = 0.437, *p* < 0.001), with coworkers (r = 0.367, *p* < 0.001), with parents (r = 0.343, *p* < 0.001).

A parallel mediation was performed to investigate the mediator role of the different sub-scales of satisfaction in the relationship between self-efficacy and well-being.

The results indicated that self-efficacy had a significant and positive total effect on well-being (path c; *β* = 0.644, *p* < 0.001; LLCI = 0.25, ULCI = 0.39). Specifically, the self-efficacy had significant and positive effects on satisfaction: satisfaction with students (path a1; *β* = 0.40, *p* < 0.001; LLCI = 0.05, ULCI = 0.09), satisfaction with coworkers (path a2; *β* = 0.14, *p* = 0.01; LLCI = 0.01, ULCI = 0.05), satisfaction with parents (path a3; *β* = 0.22, *p* < 0.001; LLCI = 0.03, ULCI = 0.07). In turn, the three sub-scales were positively and significantly related to well-being (path b1; *β* = 0.21, *p* < 0.001; LLCI = 0.43, ULCI = 1.33; path b2; *β* = 0.25, *p* < 0.001; LLCI = 0.64, ULCI = 1.33; path b3; *β* = 0.11, *p* < 0.05; LLCI = 0.02, ULCI = 0.75). The direct effect between self-efficacy and well-being is also significant and positive (path c’; *β* = 0.30, *p* < 0.001; LLCI = 0.15, ULCI = 0.29). Therefore, the results indicated partial mediation, with a significant total effect (*R*^2^ = 0.20; *F*_(1, 337)_ = 80.33, *p* < 0.001).

## 5. Discussion

The recent literature has highlighted that teachers face increasing work-related stress and burnout due to growing professional demands and organizational pressures, making the identification of protective factors for their well-being a central topic in the educational field [1,2,5,13]. The present study aimed to examine whether three dimensions of relationship satisfaction, satisfaction with students, colleagues, and parents, mediate the association between self-efficacy and well-being in a sample of Italian teachers.

The present findings provide support for Hypothesis 1, showing that teachers’ self-efficacy was positively associated with all three dimensions of satisfaction, toward students, colleagues, and parents, as well as with overall well-being, and that each satisfaction sub-scale was in turn positively related to well-being (H1). This pattern is consistent with social-cognitive theory [14], according to which efficacy beliefs sustain adaptive coping, persistence, and engagement in challenging contexts. These findings align with previous studies indicating that higher levels of teacher self-efficacy are associated with greater job satisfaction and more positive emotional and psychological outcomes. Indeed, self-efficacy has been identified as a significant antecedent of both job satisfaction and overall well-being, suggesting that higher perceived competence enhances positive emotional experiences and reduces work-related strain [38]. Self-efficacy also contributes to key adaptive resources, with evidence indicating that teachers who feel more capable of managing instructional and relational challenges exhibit stronger professional engagement and resilience [39]. Moreover, recent findings suggest that self-efficacy exerts a protective function by fostering more positive relational and emotional experiences within the school environment [40]. Taken together, these studies provide converging support for the present findings. They show that teachers who believe in their own abilities tend to feel more satisfied in their professional relationships and enjoy better mental well-being. This highlights just how crucial those beliefs in one’s effectiveness are as a personal resource in the world of education. Teachers who feel more capable are generally better at handling classroom challenges, building positive relationships with their students, working together with their colleagues, and communicating well with parents. In line with this, previous research has suggested that higher self-efficacy is associated with greater job satisfaction, stronger perceived social support, and lower levels of stress and burnout.

The mediation model further revealed that satisfaction with students, colleagues, and parents each acted as a significant mediator in the relationship between self-efficacy and well-being, while the direct effect of self-efficacy remained significant. This pattern of partial mediation provides partial support for Hypothesis 2, indicating that relational satisfaction is one important pathway through which self-efficacy contributes to well-being, but not the only one (H2). The positive impact of teachers’ self-efficacy on job satisfaction is widely documented in the literature [41]. Overall, these studies converge in showing that as teachers’ perceived self-efficacy increases, their satisfaction with work also tends to rise [42,43,44]. High efficacy beliefs appear to protect teachers from stress and burnout and to promote greater job satisfaction [45], which is especially relevant given the central role of teaching for the future of societies. As argued by Erden [46], it is difficult to obtain effective performance from teachers who are dissatisfied with their work and feel that their professional expectations are not met. Teachers’ self-efficacy beliefs predict job satisfaction, which in turn was associated with students’ academic achievement, highlighting the broader impact of these psychosocial resources [30]. While confirming that all three relational domains act as significant mediators, the present findings highlight meaningful differences in their relative strength. Satisfaction with parents showed the strongest association with well-being, followed by satisfaction with students and, to a lesser extent, satisfaction with colleagues. Such differences may reflect relational demands specific to the Italian school context. Interactions with parents often extend beyond instructional tasks and can shape teachers’ sense of validation, trust, and emotional safety, thus exerting a strong influence on their well-being [32]. Satisfaction with students may enhance well-being by fostering a sense of meaning, classroom harmony, and professional accomplishment [24,47,48]. Satisfaction with colleagues, while still important, may operate indirectly by contributing to a supportive school climate, rather than directly influencing daily emotional experiences [49]. From a practical perspective, these distinctions suggest that interventions aimed at strengthening communication and collaboration with parents, and promoting positive teacher-student relationships, may have a particularly beneficial effect on teachers’ well-being [24,32]. At the same time, enhancing collegial support remains essential for sustaining a healthy organizational climate, even if its mediating role is comparatively weaker [47,49].

More broadly, it has been suggested that well-being in teaching stems largely from the fulfillment of higher-order needs, such as deriving meaning and satisfaction from positive social relationships, which play a more significant role in our lives than just the basic incentives like salary [50]. Supporting this idea, recent studies highlight how crucial interpersonal relationships are in the teaching profession [49]. In fact, feeling satisfied with the support from colleagues, parents, and students can help mitigate some of the challenges that come with teaching, ultimately boosting overall well-being [24,47,48]. In this sense, job satisfaction emerges as a key correlate of subjective well-being [51], reinforcing the importance of fostering both self-efficacy and relational resources within school settings.

### 5.1. Limitations

Several limitations should be considered when interpreting the present findings. First, the cross-sectional design prevents drawing causal inferences among the examined variables (e.g., [52]), meaning that the observed associations between self-efficacy, relational satisfaction, and well-being cannot be interpreted in terms of directionality and may also reflect the influence of unmeasured factors. Accordingly, while the pattern of results is consistent with previous literature, it should be read as indicative of concurrent relationships rather than definitive evidence of underlying mechanisms. Future research adopting longitudinal (and, where possible, multi-wave) designs would help clarify the temporal ordering of these constructs and test whether they reciprocally influence one another over time.

Second, the study relied on self-report measures. These can sometimes be influenced by social desirability, personal interpretation, or even memory lapses. As Snow [53] pointed out, self-reported data might not fully capture the intricate nature of personal traits. To enhance the reliability and validity of future assessments, it would be beneficial to incorporate multi-method approaches, like behavioral observations or physiological indicators [54]. When it comes to sampling, the data was gathered online through convenience sampling, and participation was voluntary. This could potentially limit how broadly the findings can be applied. While the gender distribution in the sample mirrors the predominantly female makeup of the Italian teaching workforce [55], this imbalance might hinder the generalizability to more gender-diverse populations. To get a clearer picture, future research should aim to include larger and more varied samples, both culturally and socio-demographically. This would help determine if these results are consistent across different groups and settings [52]. Nonetheless, the sample size was adequate for the analyses conducted and provided sufficient statistical power for the mediation model.

The third point concerns the variables that influence teacher well-being. As shown in numerous studies, the latter is affected by a very wide range of psychological resources. However, in this study, we chose to focus primarily on the variables of self-efficacy and relational satisfaction. Recent literature highlights the contribution of several variables, such as positive affect, emotion regulation, and basic psychological need satisfaction, as central determinants of teachers’ emotional functioning. The research conducted by Moè and Katz [56] demonstrated that, in the Italian context, teachers report greater well-being when their needs for autonomy, competence, and relatedness are fulfilled and when they can rely on adaptive emotion regulation strategies. Other studies have emphasized how motivational and socio-emotional factors influence teachers’ adjustment, stress levels, and overall well-being [57], while still others have shown how psychological resources (e.g., enthusiasm, perceived support, and socio-emotional need satisfaction) sustain well-being and help reduce burnout [33,58]. Overall, this evidence suggests that the constructs outlined above may coexist with self-efficacy and relational satisfaction in predicting teacher well-being. Although these variables fall outside the scope of the present study, integrating them into future research would contribute to a more comprehensive and multidimensional understanding of the motivational and emotional processes that support teachers’ psychological health.

### 5.2. Practical Implications

Given the positive association between teachers’ self-efficacy, job satisfaction, and well-being, it appears crucial for schools to implement organizational strategies and professional development initiatives aimed at strengthening teachers’ sense of efficacy. Evidence from the recent literature shows that structured training programs, particularly those explicitly designed to enhance self-efficacy, produce significant improvements in teachers’ perceived competence, stress management, and overall occupational well-being [59,60,61]. Group-based interventions, like resilience workshops, mindfulness programs, and collaborative stress-reduction activities, have proven to be effective in lowering burnout and fostering healthier and more supportive work environments [51,62]. Moreover, initiatives that focus on relational aspects, such as teacher–student interaction programs, have been shown to strengthen classroom relationships, boost student behavior and engagement, and create a more positive and sustainable teaching experience (e.g., [63]). Additionally, enhancing collaboration between teachers and families is another important way to improve the school climate: past research suggests that structured parental involvement programs lead to better student outcomes and more supportive relational environments, which in turn can indirectly contribute to teachers’ satisfaction and well-being [64,65]. Given these insights, offering teachers regular in-service training, chances for collaborative professional learning, and structured opportunities to engage with parents could be effective strategies for boosting both their professional effectiveness and job satisfaction.

## 6. Conclusions

The present study expands the current knowledge on teachers’ well-being by examining how self-efficacy and relational satisfaction jointly contribute to psychological health in the school context. By focusing on both individual resources, and relational dimensions shaped by daily interactions with students, colleagues, and parents, our findings shed light on the mechanisms through which teachers navigate the demands of their profession. The significant indirect effects observed in the mediation model highlight the important role of relational satisfaction as a pathway linking efficacy beliefs to well-being, suggesting that teachers’ perceptions of supportive and positive relationships within the school community may effectively enhance the benefits associated with strong self-efficacy beliefs. These results align with the broader literature emphasizing the protective value of self-efficacy and positive interpersonal relationships for teachers’ professional functioning and overall satisfaction (e.g., [49]). At the same time, the complexity of these processes indicates the need for continued investigation, particularly to better understand how different relational domains uniquely shape teachers’ experiences. Overall, this study provides evidence that fostering both personal competencies and high-quality social connections may be essential for promoting sustainable well-being among teachers, offering relevant insights for future research and educational practice.

## Figures and Tables

**Table 1:** Correlations among variables.

	Well-Being	Self-Efficacy	Satisfaction with Colleagues	Satisfaction with Students
**Self-efficacy**	0.439 ***			
**Satisfaction** **with colleagues**	0.367 ***	0.140 ***		
**Satisfaction** **with students**	0.437 ***	0.402 ***	0.218 ***	
**Satisfaction** **with parents**	0.343 ***	0.225 ***	0.246 ***	0.510 ***

Note. *** *p* < 0.001.

## Data Availability

The data presented in this study are available from the corresponding author upon reasonable request.

## References

[1] Hascher T., Waber J. (2021). Teacher well-being: A systematic review of the research literature from the year 2000–2019. Educ. Res. Rev..

[2] Capone V., Joshanloo M., Park M.S.A. (2019). Burnout, depression, efficacy beliefs, and work-related variables among school teachers. Int. J. Educ. Res..

[3] De Stasio S., Fiorilli C., Benevene P., Uusitalo-Malmivaara L., Chiacchio C.D. (2017). Burnout in special needs teachers at kindergarten and primary school: Investigating the role of personal resources and work wellbeing. Psychol. Sch..

[4] Seibert E.M., Skaalvik S. (2014). Teacher self-efficacy and perceived autonomy: Relations with teacher engagement, job satisfaction, and emotional exhaustion. Psychol. Rep..

[5] Kyriacou C., Wright J.D. (2015). Teacher stress and burnout: Methodological perspectives. International Encyclopedia of the Social & Behavioral Sciences.

[6] Gabola P., Iannaccone A. (2015). Elementi contestuali nella costruzione del benessere degli insegnanti in due casi studio italiani. Schweiz. Z. Bild..

[7] Chirico F., Capitanelli I., Taino G., Rizzo A., Cramarossa A.A., Sacco A. (2024). Fattori di rischio e misure di prevenzione per gli insegnanti: Una revisione di letteratura con una proposta di protocollo di sorveglianza sanitaria nelle scuole. Giornal Ital. Psicol. Med. Lav..

[8] Johnson B., Down B., Le Cornu R., Peters J., Sullivan A., Pearce J., Hunter J. (2014). Promoting early career teacher resilience: A framework for understanding and acting. Teach. Teach..

[9] Pelletier L.G., Séguin-Lévesque C., Legault L. (2002). Pressure from above and pressure from below as determinants of teachers’ motivation and teaching behaviors. J. Educ. Psychol..

[10] Stanzione I., Calenda M. (2020). Gli effetti del burn-out e di basse percezioni del contesto lavorativo degli insegnanti sulle condizioni di benessere e disagio degli studenti: Un’indagine sulla scuola secondaria di primo grado di Roma e Salerno. Lifelong Lifewide Learn..

[11] Agyapong B., Obuobi-Donkor G., Burback L., Wei Y. (2022). Stress, burnout, anxiety and depression among teachers: A scoping review. Int. J. Environ. Res. Public Health.

[12] Hamre B.K., Justice L.M., Pianta R.C., Kilday C., Sweeney B., Downer J.T., Leach A. (2010). Implementation fidelity of MyTeachingPartner literacy and language activities: Association with preschoolers’ language and literacy growth. Early Child. Res. Q..

[13] You S., Kim A.Y., Lim S.A. (2017). Job satisfaction among secondary teachers in Korea: Effects of teachers’ sense of efficacy and school culture. Educ. Manag. Adm. Leadersh..

[14] Bandura A. (1997). Self-Efficacy: The Exercise of Control.

[15] Tschannen-Moran M., Hoy A.W. (2001). Teacher efficacy: Capturing an elusive construct. Teach. Teach. Educ..

[16] Vieira L., Rohmer O., Jury M., Desombre C., Delaval M., Doignon-Camus N., Popa-Roch M. (2024). Attitudes and self-efficacy as buffers against burnout in inclusive settings: Impact of a training programme in pre-service teachers. Teach. Teach. Educ..

[17] Zee M., Koomen H.M. (2016). Teacher self-efficacy and its effects on classroom processes, student academic adjustment, and teacher well-being: A synthesis of 40 years of research. Rev. Educ. Res..

[18] Rodríguez-Sánchez A., Salanova M., Cifre E., Schaufeli W.B. (2011). When good is good: A virtuous circle of self-efficacy and flow at work among teachers. Rev. Psicol. Soc..

[19] Capone V., Joshanloo M., Park M.S.A. (2023). Job satisfaction mediates the relationship between psychosocial and organization factors and mental well-being in schoolteachers. Int. J. Environ. Res. Public Health.

[20] Pepe A., Addimando L., Veronese G. (2017). Measuring teacher job satisfaction: Assessing invariance in the Teacher Job Satisfaction Scale (TJSS) across six countries. Eur. J. Psychol..

[21] Roffey S. (2012). Pupil wellbeing-teacher wellbeing: Two sides of the same coin?. Educ. Child Psychol..

[22] Skaalvik E.M., Skaalvik S. (2009). Does school context matter? Relations with teacher burnout and job satisfaction. Teach. Teach. Educ..

[23] Riasudeen S., Singh P., Kannadhasan M. (2019). The role of job satisfaction behind the link between group cohesion, collective efficacy, and life satisfaction. Psychol. Stud..

[24] Cano-García F.J., Padilla-Muñoz E.M., Carrasco-Ortiz M.Á. (2005). Personality and contextual variables in teacher burnout. Personal. Individ. Differ..

[25] Chang M.L. (2009). An appraisal perspective of teacher burnout: Examining the emotional work of teachers. Educ. Psychol. Rev..

[26] Veldman I., Van Tartwijk J., Brekelmans M., Wubbels T. (2013). Job satisfaction and teacher-student relationships across the teaching career: Four case studies. Teach. Teach. Educ..

[27] Day C., Gu Q. (2013). Veteran teachers: Commitment, resilience and quality retention. International Perspectives on Veteran Teachers.

[28] Ghenghesh P. (2013). Job satisfaction and motivation: What makes teachers tick?. Br. J. Educ. Soc. Behav. Sci..

[29] Jeynes W.H. (2010). The salience of the subtle aspects of parental involvement and encouraging that involvement: Implications for school-based programs. Teach. Coll. Rec..

[30] Caprara G.V., Barbaranelli C., Steca P., Malone P.S. (2006). Teachers’ self-efficacy beliefs as determinants of job satisfaction and students’ academic achievement: A study at the school level. J. Sch. Psychol..

[31] Capone V., Petrillo G. (2020). Mental health in teachers: Relationships with job satisfaction, efficacy beliefs, burnout and depression. Curr. Psychol..

[32] Aldridge J.M., Fraser B.J. (2016). Teachers’ views of their school climate and its relationship with teacher self-efficacy and job satisfaction. Learn. Environ. Res..

[33] Avanzi L., Fraccaroli F., Castelli L., Marcionetti J., Crescentini A., Balducci C., van Dick R. (2018). How to mobilize social support against workload and burnout: The role of organizational identification. Teach. Teach. Educ..

[34] Biasi V., Domenici G., Patrizi N., Capobianco R. (2014). Teacher Self-Efficacy Scale (Scala sull’auto-efficacia del docente-SAED): Adattamento e validazione in Italia. J. Educ. Cult. Psychol. Stud. (ECPS J.).

[35] Keyes C.L.M. (2002). The mental health continuum: From languishing to flourishing in life. J. Health Behav. Res..

[36] Petrillo G., Capone V., Caso D., Keyes C.L. (2015). The Mental Health Continuum-Short Form (MHC-SF) as a measure of well-being in the Italian context. Soc. Indic. Res..

[37] Hayes A.F. (2018). Partial, conditional, and moderated moderated mediation: Quantification, inference, and interpretation. Commun. Monogr..

[38] Ortan F., Simut C., Simut R. (2021). Self-efficacy, job satisfaction and teacher well-being in the K-12 educational system. Int. J. Environ. Res. Public Health.

[39] Heng Q., Chu L. (2023). Self-efficacy, reflection, and resilience as predictors of work engagement among English teachers. Front. Psychol..

[40] Wang X., Gao Y., Wang Q., Zhang P. (2024). Relationships between self-efficacy and teachers’ well-being in middle school English teachers: The mediating role of teaching satisfaction and resilience. Behav. Sci..

[41] Soto M., Rojas O. (2019). Self-efficacy and job satisfaction as antecedents of citizenship behaviour in private schools. Int. J. Manag. Educ..

[42] Arslan G. (2019). School belonging in adolescents: Exploring the associations with school achievement and internalising and externalising problems. Educ. Child Psychol..

[43] Demir S. (2020). The role of self-efficacy in job satisfaction, organizational commitment, motivation and job involvement. Eurasian J. Educ. Res..

[44] Won S.D., Chang E.J. (2020). The relationship between school violence-related stress and quality of life in school teachers through coping self-efficacy and job satisfaction. Sch. Ment. Health.

[45] OECD (2020). OECD Family Database.

[46] Erden M. (2007). Eğitim Bilimlerine Giriş [Introduction to Educational Sciences].

[47] Skaalvik E.M., Skaalvik S. (2011). Teacher job satisfaction and motivation to leave the teaching profession: Relations with school context, feeling of belonging, and emotional exhaustion. Teach. Teach. Educ..

[48] Gavish B., Friedman I.A. (2010). Novice teachers’ experience of teaching: A dynamic aspect of burnout. Soc. Psychol. Educ..

[49] Van Droogenbroeck F., Spruyt B., Vanroelen C. (2014). Burnout among senior teachers: Investigating the role of workload and interpersonal relationships at work. Teach. Teach. Educ..

[50] Sylvia R.D., Hutchison T. (1985). What makes Ms. Johnson teach? A study of teacher motivation. Hum. Relat..

[51] Seibert S.E., Kraimer M.L., Liden R.C. (2001). A social capital theory of career success. Acad. Manag. J..

[52] De Beer L.T., Schaufeli W.B., De Witte H., Hakanen J.J., Shimazu A., Glaser J., Rudnev M. (2020). Measurement invariance of the Burnout Assessment Tool (BAT) across seven cross-national representative samples. Int. J. Environ. Res. Public Health.

[53] Snow N.E. (2019). Positive psychology, the classification of character strengths and virtues, and issues of measurement. J. Posit. Psychol..

[54] Karris Bachik M.A., Carey G., Craighead W.E. (2021). VIA character strengths among US college students and their associations with happiness, well-being, resiliency, academic success and psychopathology. J. Posit. Psychol..

[55] OECD (2018). TALIS 2018 Results (Volume I): Teachers and School Leaders as Lifelong Learners.

[56] Moè A., Katz I. (2022). Need satisfied teachers adopt a motivating style: The mediation of teacher enthusiasm. Learn. Individ. Differ..

[57] Costa A., Moreira D., Casanova J., Azevedo Â., Gonçalves A., Oliveira Í., Azevedo R., Dias P.C. (2024). Determinants of academic achievement from the middle to secondary school education: A systematic review. Soc. Psychol. Educ..

[58] Fiorilli C., Farina E., Buonomo I., Costa S., Romano L., Larcan R., Petrides K.V. (2020). Trait emotional intelligence and school burnout: The mediating role of resilience and academic anxiety in high school. Int. J. Environ. Res. Public Health.

[59] Täschner J., Dicke T., Reinhold S., Holzberger D. (2025). “Yes, I can!” A systematic review and meta-analysis of intervention studies promoting teacher self-efficacy. Rev. Educ. Res..

[60] Beames J.R., Spanos S., Roberts A., McGillivray L., Li S., Newby J.M., Werner-Seidler A. (2023). Intervention programs targeting the mental health, professional burnout, and/or wellbeing of school teachers: Systematic review and meta-analyses. Educ. Psychol. Rev..

[61] Avola P., Soini-Ikonen T., Jyrkiäinen A., Pentikäinen V. (2025). Interventions to teacher well-being and burnout: A scoping review. Educ. Psychol. Rev..

[62] Hidajat T.J., Edwards E.J., Wood R., Campbell M. (2023). Mindfulness-based interventions for stress and burnout in teachers: A systematic review. Teach. Teach. Educ..

[63] Borgonovi F. (2015). Do Teacher-Student Relations Affect Students’ Well-Being at School?.

[64] Jeynes W.H. (2011). Parental involvement research: Moving to the next level. Sch. Community J..

[65] Jeynes W.H. (2012). A meta-analysis of the efficacy of different types of parental involvement programs for urban students. Urban Educ..

